# Maternal education, anthropometric markers of malnutrition and cognitive function (ELSA-Brasil)

**DOI:** 10.1186/1471-2458-14-673

**Published:** 2014-07-02

**Authors:** Larissa Fortunato Araújo, Luana Giatti, Dora Chor, Valéria Maria Azeredo Passos, Sandhi Maria Barreto

**Affiliations:** 1 Research Group on Epidemiology of Chronic Diseases (GERMINAL), Faculty of Medicine, Universidade Federal de Minas Gerais, Minas Gerais, Brazil; 2 School of Nutrition, Universidade Federal de Ouro Preto, Minas Gerais, Brazil; 3 National School of Public Health, Fundação Oswaldo Cruz, Rio de Janeiro, Brazil

**Keywords:** Maternal education, Nutritional markers, Life course, Cognitive function

## Abstract

**Background:**

The early exposure to poor social and nutritional conditions may influence cognitive function during adult age. However, the relative impact of these factors has not yet been established and they can vary during the course of life.

**Methods:**

Analysis of data from 12,997 participants (35-64 years) of the baseline exams (2008-2010) of the Longitudinal Study of Adult Health (ELSA-Brasil), a cohort of Brazilian civil servants. Four cognitive tests were applied: learning, recall and word recognition; semantic and phonemic verbal fluency; trail-making test version B. The markers of early nutritional and social conditions were maternal educational level, birth weight, and length of trunk and leg. The presence of independent association between every early marker and the poor performance in each cognitive test was investigated by multiple logistic regression, after mutual adjustment and considering the effects of gender, age and participant’s schooling level. The cut off for poor performance was the worst age-specific percentile of the final score distribution for each test.

**Results:**

After full adjustments, lower maternal education increased the chances of poor performance in all cognitive tests, with a dose-response gradient; low birth-weight was related to poor performance in the trail-making test B (OR = 1.63, 95% IC = 1.29-2.06); and greater trunk length decreased the chances of poor performance in the semantic and phonemic verbal fluency (OR = 0.96, 95% IC = 0.94-0.97) and in the trail-making test B (OR = 0.94, 95% IC = 0.92-0.95). Leg length was not associated with any of the tests examined. The associations found were not modified by the educational attainment of the participants.

**Conclusions:**

Early exposure to adverse social and nutritional conditions appear detrimental to semantic memory, learning, concentration, executive control and language among adults, independent of adulthood educational achievement.

## Background

Lifetime cognitive development results from a combination of normative and non-normative processes that affect cerebral function [1]. Normative ageing is associated with better cognitive abilities during early adulthood, followed by a period of relative cognitive stability during adulthood and cognitive decline with advanced age. Non-normative ageing refers to effects that go beyond the normative process and result in rapid cognitive decline, even before a disease such as Alzheimer’s is diagnosed [2]. Investigating the factors that influence cognitive function during adulthood is essential in order to understand these two ageing processes.

Children from families with low socioeconomic position (SEP) have much greater chances of worse health and psychological well-being, as well of impaired cognitive and emotional development throughout the lifespan [3]. Many indicators of SEP in the childhood have been linked to poor cognitive outcomes among adult and older individuals [4-6]. Kaplan et al. [7] observed that children of mothers with higher levels of education had better cognitive function during adulthood. Lower socioeconomic status during childhood was independently associated with worse semantic memory and increased rate of cognitive decline in later life among an ethnically diverse cohort in the US [8]. The influence of lifetime SEP on cognitive ageing is particularly important to low and middle income countries due to the enduring and profound social inequalities as well the high prevalence of poverty that prevails in most of these societies.

Victora et al. [9] showed that maternal and child malnutrition during the first year of life can cause irreversible damages, including shorter adult height, lower educational level and reduced adult income, and lower birth weight of offspring. Early life factors, such as low birth weight and/or growth problems during childhood and puberty seem also to have adverse effects on cognitive function in adulthood [7,10-12]. Lower adult height, which can indicate bad nutritional conditions during childhood, has been associated with worse cognitive function in adults [13-15].

There is considerable debate concerning the mechanisms by which adverse SEP and nutritional conditions during early life stages influences cognitive function later in life [16]. Hackman et al. [3] listed three potential mechanisms underlying the effects of SEP’s on neurocognitive development: prenatal factors, parental care and cognitive stimulation. The prenatal period is a sensitive time during which intrauterine exposures can alter the course of development with enduring effects on the offspring. Review of findings from clinical, epidemiologic, and basic science research indicates that poor maternal diet and psychosocial distress during pregnancy can significantly affect children’s future neurodevelopment, especially memory related [10]. Low SEP is associated with parental irritability, depression and anxiety and can compromise the parent– child interactions [17]. In a longitudinal study, parental care in early childhood predicted better declarative memory and smaller hippocampal volume in low SEP adolescents, independent of cognitive stimulation and maternal intelligence [17].

Epigenetic theory postulates that the mismatch between the maternally programmed behaviors of the offspring, and the actual environment in which he develops, may create disadvantages that increase the offspring susceptibility to health problems in adulthood, including those related to stress response, memory, and cognitive capacity [18]. Cognitive stimulation during childhood is very important to boost academic and professional achievement, which are strong predictors of cognitive function in adulthood [7,19]. Differences in the quality and quantity of schooling is one plausible mechanism for the differences in cognitive function related to SEP, but a review on this subject showed that achievements in formal education cannot, on their own, account for all of the variance in cognition and brain development attributable to SEP [20].

Low- and medium-income countries (LMIC) are facing an accelerated ageing process [21], and cognitive impairment has become a growing public health problem. Currently, large numbers of people with dementia live in LMIC and rapid increases are predicted in the near future [22]. However, few studies have examined the long lasting effects of early life SEP and nutritional conditions on cognitive ageing in these countries [11,23,24]. Despite recent socioeconomic improvements, the greater exposure to adverse conditions during pregnancy and childhood of most Brazilians in the 20^th^ century makes Brazilians quite different from populations of high-income countries. Thus, even a small effect of an exposure to early adverse SEP on adult cognitive function is likely to have greater impact on the Brazilian population as a whole, due to the higher prevalence of such adversities in the country.

The present study investigates whether the exposure to adverse socioeconomic and nutritional conditions early in life influences cognitive function in adulthood, using the baseline data from the Longitudinal Study of Adult Health (ELSA-Brasil).

## Methods

The ELSA-Brasil is a prospective multicenter study with 15,105 active and retired civil servants of higher education and research institutions in six Brazilian states. A detailed description of the baseline study has been published elsewhere [25,26].

This study uses data regarding all the adults (35-64 years old) who undertook cognitive tests in the baseline study (2008-2010). Since intrinsic aspects of ageing and certain clinical conditions affect cognitive function, participants aged 65 years or more and those who reported previous diagnosis of stroke and/or who were using neuroleptics, anticonvulsants, anticholinesterase or antiparkinsonian drugs did not participate in the present analysis. We also excluded participants who had reading difficulties and those who took the learning, recall and word recognition tests using figures instead of words.

From 12,997 eligible participants, 12,988 took the learning, recall and word recognition test (99.9%), 12,953 the semantic and phonemic verbal fluency tests (99.7%) and 12,399 the trail-making test B (95.4%). All participants had their standing and sitting height measured; 10,707 knew their birth weight and were term birth (86.9%), and 12,742 reported their mothers’ educational level (98.0%).

### Response variables

The response variables were the final scores obtained in the following cognitive function tests:

1) The learning, recall and word recognition tests of the Consortium to Establish a Registry for Alzheimer’s Disease (CERAD) [27], validated for the elderly Brazilian population, were used to evaluate declarative memory. We used the simple sum of the scores of these three tests.

2) The semantic (animal category) and phonemic (letter F) verbal fluency tests, which are also part of the CERAD’s [27] group of tests, were used to evaluate semantic declarative memory and language. The score corresponds to the total number of correct animal names and words beginning with the letter “F” given by the participant. Initially, the semantic verbal fluency (animals) and the phonemic verbal fluency (letter F) tests were analyzed separately, but there were no differences in the direction and magnitude of the associations found. For this reason, they were after analyzed together and we considered the simple sum of the scores of both tests.

3) The trail-making test evaluates executive function, which is related to attention, concentration and psychomotor speed [28]. The Trail-making test version A was used as a training test by the participants. The score corresponded to the time (in seconds) taken to complete trail-making test version B. In this article, we will refer to the trail-making test version B such as trail-making test B.

The reliability of these tests varied from moderate, for the learning and word recall test (Kappa = 0.56; 0.33-0.79), to very good, for the trail making test B (Kappa = 0.91; 0.87-0.95) [29].

The present study did not use predefined cut-off points, as they are not available for healthy and functional adults. The final score obtained in each test was categorized using age-specific deciles based on 5-year age intervals (35-39, 40-44, 45-49, 50-54, 55- 59, 60-64). The individuals located in the first decile of the learning, recall and word recognition tests and of the semantic and phonemic verbal fluency tests and those located in the tenth decile of the trail-making test B were regarded as having poor test performance. The age-specific cut-off points that defined poor performance in each test were presented in the Table 1.

**Table 1:** Age-specific cut-off points that represented poor performance in the learning, recall and word recognition tests, semantic and phonemic verbal fluency tests and trail-making test B among adults’ participants of the ELSA-Brasil (2008-2010)

	**Learning, recall and word recognition (n = 12,988)**	**Semantic and phonemic verbal fluency (n = 12,593)**	**Trail-making test B (n = 12,399)**
**Age (years)**	**Up to (no. of words)**	**Up to (no. of words)**	**Above (duration in seconds)**
*35-39*	34	24	129
*40-44*	32	23	153
*45-49*	31	22	175
*50-54*	30	20	225
*55-59*	28	19	256
*60-64*	28	19	266

### Explanatory variables of interest

The level of maternal educational attainment was assessed by the question, “What is your mother’s educational level?” and the answers grouped into four categories: high school or more (≥11 years of school), complete elementary school (≥8 and <11 years of school); did not finish elementary school (<8 years of school); and never attended school. The birth weight was obtained by the question: “According to the information you have, what was your birth weight?” and a weight inferior to 2.5 kg was classified as being low birth weight. The participants who reported premature birth were not included in this analysis, because prematurity affects not only birth weight but also can compromise growth development beyond birth weight.

Since leg length and trunk length are influenced by nutritional conditions in different phases of a child development, we used both measures to investigate the relationship between nutritional conditions in early life and cognitive function in adulthood [30]. Leg and trunk length (in centimeters) were ascertained by combining the standing and the sitting heights. Trunk length was obtained by subtracting the height of the bench (46 cm) used to measure the sitting height from the total sitting height. Leg length was obtained by subtracting the trunk length from the standing height. In order to avoid multicolinearity with age, for the regression analysis we created centered variables for leg and trunk length by means of subtracting each individual measure of leg and trunk lengths from their gender-specific means. The centered leg and trunk length were entered as continuous variables in the analysis.

### Adjusting variables

The following variables were considered as potential confounders in the analyses: gender, age (continuous in the multivariate analyses) and current educational level (undergraduate school, high school, elementary school, incomplete elementary school). Income per capita was not included as a control variable due to its high correlation with educational attainment (Spearman’s rho = 0.69).

### Statistical analysis

The associations between the covariates and each response variable were estimated by the chi-square test (categorical variables) and by the t test for means (continuous variables) using a significance level of p < 0.05. The magnitude of the associations were estimated by the odds ratio (OR) and its 95% confidence interval, obtained by logistic regression.

The spearman correlation coefficients regarding the explanatory variables of interest were weak, except for the one concerning leg and trunk lengths, which was moderate (data not shown). We tested for possible multicollinearity between the variables included in the final multivariate model by calculating the variance inflation factor (VIF).

Due to the high percentage (13.1%) of missing information on birth weight, we performed the multiple imputation procedure by logistic regression to correct for this loss of information, using the following variables in the estimation of missing data: gender, age, education, leg and trunk lengths, and maternal education. We assumed that missing data on birth weight was not because of any specific variable for which information was lost. Each missing information was imputed twenty times, given its binary nature and the large amount of missing data. A complete data analysis was performed on each imputed data set, and their final results were combined according to the rule of Rubin (1987) [31]. The imputation was performed using the package MICE [32] of the R software version 3.0.1. The birth weight was imputed for the participants with term birth, and the imputed variable was used only in the analysis by logistic regression.

We guided the multivariate analysis by the life-course approach, which hypothesizes that the health in adulthood is affected by the complex interplay of social and biological factors during pregnancy, infancy and childhood [33]. A fully adjusted OR for the association between every explanatory variable of interest (maternal education, birth weight, trunk and leg lengths) and poor performance in each of the cognitive tests was obtained by multivariate logistic regression analysis. Firstly, we estimated the crude OR for each exposure of interest (Model 0), then we adjusted the crude OR that were statistically significant for age, gender and participant’s schooling level (Model 1). Afterwards, we further adjusted the OR for the other exposures of interest (Model 2), whenever they were also associated with the response variable at the level of p < 0.05 in Model 1. We retained in the final model all the variables that remained statistically associated with the response variable (p < 0.05) after all the adjustments. In order to test for possible heterogeneity in the effect of every variable of interest according to the educational attainment of the participant we created and added interaction terms to the final model, retaining the ones that were statistically significant. The goodness of fit of the final models was assessed by the Hosmer & Lemeshow test. The analysis was performed using Stata 12.0 (Stata Corporation, College Station, USA)

### Ethical aspects

All participants signed a free and informed consent form. The study was approved by the National Committee of Ethics in Research (approval no 976/2006).

## Results

Among the 12,997 participants, 54.8% were women, 25.2% were between the age of 35 and 44 years, 44.0% between 45 and 54 years, and 30.8% between 55 and 64 years. Most of the participants (52.7%) had undergraduate education, 36.5% had completed graduate school, and 4.4% had not finished elementary school. The majority of the participants’ mothers did not complete elementary school (56.2%) and 24.4% had high school or more. The frequency of low birth weight was 5.5% (Table 2).

**Table 2:** Socio-demographic characteristics and birth weight of participants of the ELSA-Brasil (2008-2010)

**Variables**	**N**	**%**
*Gender*		
Male	5,880	45.2
Female	7,117	54.8
*Age (years)*		
34-39	1,133	8.7
40-44	2,145	16.5
45-49	3,006	23.1
50-54	2,716	20.9
55-59	2,430	18.7
60-64	1,567	12.1
*Educational attainment*		
Undergraduate school or more	6,851	52.7
High school	4,743	36.5
Elementary school	826	6.4
Incomplete elementary school	577	4.4
*Maternal education*		
High school or more	3,112	24.4
Elementary school	2,471	19.4
Incomplete Elementary school	5,492	43.1
Never attended school	1,667	13.1
Missing	255	2.0
*Birth-weight (Kg)*		
≥2.5	10,115	94.5
<2.5	592	5.5
Missing	1,621	13.1

The mean leg length was 82.34 cm (SE = 0.06) for men and 75.32 cm (SE = 0.05) for women. The mean trunk length was 90.17 cm (SE = 0.05) for men and 84.06 cm (SE = 0.04) for women.

The prevalence of poor performance in all cognitive tests was more frequent among men and reduced as the educational level of the participant increased (p < 0.001 in all tests). The frequency of poor performance was also more frequent as maternal education decreased and it was greater among participants who reported low birth weight (p < 0.001 in all tests) (Table 3). Overall, the greater leg and trunk lengths, lower the chance of poor performance in the semantic and phonemic fluency and trail-making test B (p < 0,001). The association between leg and trunk length and poor outcome in the learning, recall and word recognition test was statistically significant among men (p = 0.016), but borderline among women (p < 0.054) (Table 4).

**Table 3:** Prevalence of poor performance in the learning, recall and word recognition tests, semantic and phonemic verbal fluency tests and trail-making test B, according to socio-demographic characteristics and birth weight among adults (35-64 years-old) participants of the ELSA-Brasil (2008-2010)

**Explanatory variable**	**Learning, recall word recognition (%)**	**Semantic and phonemic verbal fluency (%)**	**Trail-making test B (%)**
*Gender*			
Male	16.8	13.0	11.6
Female	7.4	9.5	8.6
*Educational attainment*			
Under graduate school or more	5.4	3.1	2.0
High school	15.1	14.6	14.7
Elementary school	25.9	34.7	31.8
Incomplete elementary school	37.7	43.6	51.7
*Maternal education*			
High school or more	6.3	3.8	2.4
Elementary school	8.2	8.4	6.9
Incomplete Elementary school	12.3	12.0	10.5
Never went to school	22.2	23.3	26.5
*Birth-weight (Kg)*			
≥ 2.5	10.8	9.7	8.5
< 2.5	14.9	14.5	17.9

Chi-squared test with p < 0.001 in all tests.

**Table 4:** Poor performance in the learning, recall word recognition tests, semantic and phonemic verbal fluency tests, and trail-making test B, according to the leg and trunk lengths among adult men and women (35 to 64 years of age) participating in the ELSA-Brasil (2008-2010)

**Explanatory variable**	**Learning, recall word recognition**		**Semantic and phonemic verbal fluency**		**Trail-making test B**	
	**(N = 12,980)**		**(N = 12,945)**		**(N = 12,392)**	
	**Poor performance**		**Poor performance**		**Poor performance**	
	**Yes**	**No**	**Yes**	**No**	**Yes**	**No**
	**Mean (SE)**	**Mean (SE)**	**Mean (SE)**	**Mean (SE)**	**Mean (SE)**	**Mean (SE)**
*Leg length (cm)*						
Men	82.02 (0.14)^*^	82.40 (0.07)	81.56 (0.17)	82.45 (0.06)	81.72 (0.18)	82.49 (0.07)
Women	74.98 (0.17)^(a)^	75.35 (0.05)	74.79 (0.16)	75.38 (0.05)	74.82 (0.17)	75.40 (0.06)
*Trunk length (cm)*						
Men	89.54 (0.12)	90.31 (0.05)	88.80 (0.14)	90.39 (0.05)	88.47 (0.14)	90.52 (0.05)
Women	83.19 (0.15)	84.13 (0.04)	82.71 (0.14)	84.20 (0.04)	82.60 (0.14)	84.27 (0.04)

T test with p < 0.001 in all tests. ^(a)^Was not significant. ^*^0.01 < p < 0.05.

In the logistic regression analysis (Table 5), after considering the effect of gender, age and individual’s educational attainment (Model 1), lower maternal education remained associated with poor performance in all cognitive function tests. Low birth-weight continued to be associated with poor performance in the trail-making test B (OR = 1.73, 95% IC = 1.37-2.18). Leg length did not remain associated with poor performance in any of the cognitive tests examined. The OR for the association between leg length and poor outcome in the semantic and the phonemic verbal fluency test was borderline (p < 0.057). Greater trunk length decreased the chance of poor performance in the semantic and phonemic verbal fluency test (OR = 0.95, 95% IC = 0.93-0.96) and in the trail-making test B (OR = 0.93, 95% IC = 0.91-0.94). The associations between trunk length and poor performance in learning recall and word recognition test became borderline (p < 0.053). In all of the previous analysis, the associations were slightly weaker after considering the effect of the socio-demographic variables.

**Table 5:** **Crude and adjusted *Odds Ratios *** **for poor performance in the learning, recall, word recognition tests, semantic and phonemic verbal fluency tests and trail-making test B, according to maternal education, low birth-weight, leg and trunk lengths among men and women (35 to 64 years of age) participating in the ELSA-Brasil (2008-2010)**

**Cognitive function test**	**Learning, recall and word recognition**			**Semantic and phonemic verbal fluency**			**Trail-making test B**		
			**Model 2**			**Model 2**			**Model 2**
	**Crude OR**	**Model 1**	**N = 12,734**	**Crude OR**	**Model 1**	**N = 12,694**	**Crude OR**	**Model 1**	**N = 11,547**
**Explanatory variable**	**(CI-95%)**	**(CI-95%)**	**(CI-95%)**	**(CI-95%)**	**(CI-95%)**	**(CI-95%)**	**(CI-95%)**	**(CI-95%)**	**(CI-95%)**
*Maternal education*									
High school or more	1.00	1.00	1.00	1.00	1.00	1.00	1.00	1.00	1.00
Elementary school	1.32 (1.08, 1.62)^**^	1.02 (0.83, 1.27)	1.02 (0.83, 1.27)	2.34 (1.86, 2.95)^**^	1.48 (1.16, 1.89)^**^	1.42 (1.12, 1.82)^**^	3.02 (2.29, 4.00)^**^	1.80 (1.35, 2.41)^**^	1.72 (1.27, 2.33)^**^
Incomplete elementary school	2.11 (1.79, 2.49)^**^	1.36 (1.13, 1.62)^**^	1.36 (1.13, 1.62)^**^	3.46 (2.83, 4.25)^**^	1.64 (1.32, 2.03)^**^	1.58 (1.27, 1.95)^**^	4.76 (3.72, 6.09)^**^	2.07 (1.59, 2.69)^**^	1.95 (1.48, 2.56)^**^
Never went to school	4.23 (3.51, 5.08)^**^	1.89 (1.53, 2.34)^**^	1.89 (1.53, 2.34)^**^	7.76 (6.24, 9.64)^*^	2.17 (1.70, 2.78)^**^	2.02 (1.58, 2.58)^**^	14.60 (11.28, 18.90)^**^	3.89 (2.93, 5.16)^**^	3.53 (2.63, 4.75)^**^
*Birth-weight (Kg)*									
≥ 2.5	1.00	1.00	-	1.00	1.00	-	1.00	1.00	1.00
< 2.5	1.34 (1.08, 1.67)^**^	1.07 (0.85, 1.35)		1.55 (1.25, 1.92)^**^	1.13(0.90, 1.43)		2.20 (1.79, 2.71)^**^	1.73 (1.37, 2.18)^**^	1.63 (1.29, 2.06)^**^
*Centered leg length (cm)*	0.98 (0.97, 0.99)^**^	1.00 (0.99, 1.01)	-	0.96 (0.95, 0.97)^**^	0.99 (0.97, 1.00)	-	0.97 (0.95, 0.98)^**^	0.99 (0.98, 1.01)	-
*Centered trunk length (cm)*	0.94 (0.93, 0.96)^**^	0.98 (0.97, 1.00)	-	0.89 (0.88, 0.91)^**^	0.95 (0.93, 0.96)^**^	0.96 (0.94, 0.97)^**^	0.87 (0.86, 0.88)^**^	0.93 (0.91, 0.94)^**^	0.94 (0.92, 0.95)^**^

Model 1: Variables adjusted for gender, age and educational attainment of the participants. Model 2: Variable(s) that remained statistically associated after adjustment for all the variables in Model 1 plus the other explanatory variables in the table. ^*^0.01 < p < 0.05 and ^**^p < 0.01.

In Model 2 (Table 5), after full adjustment, low maternal educational level remained associated with higher chances of having a poor performance in all cognitive tests. Low birth weight only remained associated with poor performance in the trail making test B (OR = 1.63, 95% IC = 1.29-2.06). Greater trunk length persisted associated with lower chances of poor outcome in the semantic and phonemic verbal fluency tests (OR = 0.96, 95% IC = 0.94 = 0.97) and in the trail-making test B (OR = 0.94, 95% IC = 0.92- 0.95). Leg length did not remain statistically associated with poor performance in any of the cognitive tests examined. There was no statistically significant interaction between any of the explanatory variables in final models and the educational achievement of the participants. The variance inflation factor of the final models ranged from 1.71 to 3.23. The Hosmer & Lemeshow test indicated good fitting of all final models (p-values range from 0.11 to 0.84).

## Discussion

In this cohort of Brazilian public servants, we found that poor performance in all cognitive function tests was associated with lower maternal educational attainment. We also verified that participants with adverse nutritional markers at birth and during childhood exhibited higher chances of poor outcomes in the semantic and phonemic verbal fluency and in the executive function tests. We found no indication that the educational attainment of the participants modified the associations observed.

Longitudinal studies among adults have shown strong associations between early socioeconomic factors and cognitive function [34], in special between better socioeconomic conditions and better cognitive performance and slower age-related decline in cognitive performance [35,36]. However, it is still not clear which specific domains of adult cognition are associated with early socioeconomic conditions and to what extent current socioeconomic conditions, especially education, can mitigate or overcome the effects of early socioeconomic adversity that were found in this and earlier studies. Evidences suggest that childhood socioeconomic status is particularly important to performances on language and executive function tests [20].

Our findings support previous ones, which indicate that the influence of maternal education on cognitive function tend to persist during adulthood after considering the formal education of the participants. Similar association was found in a cohort of men between the age of 50 and 64 years [7]. Results from the Aging, Demographics and Memory Study [37] suggested that, after adjusting for paternal education, exposure to low maternal schooling doubled the risk of cognitive impairment, even for the elderly.

Maternal education is associated with better health and nutritional conditions during pregnancy and after birth [38,39], which indirectly predicts better health and development throughout life [9]. Higher maternal education also relates to better learning environments in childhood, more intellectual stimulation and greater mentorship quality, which, in turn, can lead to higher educational attainment and better cognitive performance [40-42]. Among the participants in this study, maternal schooling was moderately correlated with the individual’s educational level (Spearman’s rho = 0.43, data not shown).

Executive functions refer to the cognitive processes that are necessary for concentration, working memory, behavioral regulation and academic performance [43]. In the present study, the magnitude of the association between low birth weight and poor performance in the trail-making test B decreased after controlling for the effects of participant educational level, which suggests that educational attainment throughout life may mitigate the negative effects of low birth weight on adult executive function. In the Helsinki Birth Cohort, low birth weight and smaller head circumference were associated with poorer cognitive ability among men with an average age of 67.9 years and with a decline in cognitive ability after the age of 20.1 years [44].

Adult leg length is a marker of environmental and nutritional conditions during early childhood, a period of faster leg growth. In contrast, trunk length is a marker of factors taking place after early infancy and before puberty, when the trunk growth more rapidly [45,30]. A birth cohort study in Brazil showed that leg and trunk length contribute almost equally to differences in overall height, and that both biological and socioeconomic variables strongly influence determinants of height, though socioeconomic factors appear to be more important in early growth [46].

Growth considered as the reflection of genetics, childhood nutrition, and childhood medical illness, appears to provide a milieu upon which cognitive ageing occurs [8]. Many epidemiological studies reported on the association between shorter adult height and poor cognition in adults [13,15,23,47,48], but few studies have examined the separate contributions of the trunk and the lower limb.

In the present study, leg length was not associated with poor performance in any of the cognitive function tests. Increased trunk length, on the other hand, reduced the chances of poor performance in the semantic and phonemic verbal fluency tests and in the trail-making test B. That is, after adjusting for social and demographic factors, maternal education and birth weight (Model 2), there were more subjects with poor cognitive performance among participants with shorter trunk length than among those with lengthier trunks.

Heys et al. [49] observed a stronger effect of the trunk length than the leg length on cognition, suggesting that early childhood growth might be less important than later childhood development, or its effect on adult cognition more difficult to be overcome. However, a study of community residents in Korean aged 65 years and more found that only limb length (but not trunk length) was associated with dementia/cognitive impairment, after adjustment for age and education [13]. Research with healthy populations indicates that the environment is a more powerful force influencing height and body proportions than genes [44], and that parental heights have stronger influence on leg than trunk length [50]. However, as we have no information on parental height, we cannot discard the possibility of a common genetic pathway underlying both trunk growth and poor cognition in the present study.

Our results may have important implications for the Brazilian population because a sizeable fraction of the adults and elderly in the country were born from mothers with low level of education and experienced socioeconomic hardship during childhood and adolescence. More than 50% of the Brazilians were illiterate in the 1940s and 1950s, and more than one-third of the population was still illiterate in the 1970s [51]. In the 1970s, 54% of the Brazilians lived below the poverty line (per capita monthly income below US$90) and 18.6% of the children under 5 years old were malnourished [52]. Thus, Brazil will continue to have cohorts of adults and elders exposed raised by mothers with low educational attainment or who faced nutritional scarcity in childhood for many decades to come. Thus, even small effects of such exposures are of great importance to public health.

ELSA-Brasil is a longitudinal study that will evaluate cognitive function in Brazilian adults. The longitudinal design will enable the measurement of changes in cognitive function during the follow-up visits and to examine whether maternal education, birth weight, leg and trunk lengths predict cognitive decline. Despite the cross-sectional nature of these analyses, there is no doubt that the indicators of early socioeconomic position examined precede the measures of cognitive function.

The prevalence of the exposures examined in the present study is likely to differ from that found in the Brazilian population as a whole, because the ELSA-Brasil cohort is not representative of the country population, does not include the unemployed and has a much higher percentage of people with university degree. However, the social and regional diversity of the ELSA-Brasil cohort is large enough to allow the investigation of important inequities in health in Brazil [53,54], such as the ones examined in this work. As extensively debated recently, sampling representativeness is necessary when we aim to estimate the prevalence of a condition in a given population, which is not the objective of the present study, but it is not required to draw valid scientific inferences for associations found in well-conducted epidemiological studies [55,56].

We believe that the associations found in this study are not confounded by disease status as we have excluded individuals with previous history of stroke or who were using medication for neurologic or psychiatric conditions that are known to interfere with the performance on cognitive tests. The influence of maternal education, birth weight and trunk length on cognition found is not confounded by current markers of socioeconomics condition presented in this paper. However, it is possible that other early socioeconomic factors such as paternal schooling might also play some role in the associations found, but it is not available in ELSA-Brasil.

Some participants may have incorrectly informed their birth weight or mother education, but there is no reason to suppose that such misclassifications biased the associations found with poor cognitive performance. Because the percentage of missing was high for low birth weight, we performed multiple imputations for this variable, so that the final OR (and 95% CI) reported for low birth weight take into account the uncertainty due to the missing data values [57]. We also conducted complete case analysis considering only the participants who had information on all the variables considered in the analysis, and the results were similar those reported here.

## Conclusion

The present study found that the exposure to unfavorable socioeconomic and nutritional conditions during early life, represented here by low maternal educational level, low birth weight and smaller trunk length, have independent negative effects on semantic memory, learning, attention, executive control and language in a cohort of Brazilian adults. Our results suggest that educational attainment in adulthood reduces, but does not remove, the associations between worse SEP during childhood and poor cognitive performance, especially in the executive function test.

## Competing interests

The authors declare that they have no competing interests.

## Authors’ contributions

The authors SMB, LG and LFA conducted the literature review, designed the study’s analytic strategy and prepared the first and final version of the manuscript. VMAP and DC contributed to the discussion and to the final version of the manuscript. The ELSA investigators VMAP, LG and SMB implemented and evaluated cognitive function tests in ELSA-Brasil, including quality assurance and control, supervision of field activities, data processing and treatment. All authors read and approved the final manuscript.

## Pre-publication history

The pre-publication history for this paper can be accessed here:

http://www.biomedcentral.com/1471-2458/14/673/prepub
